# Phase I Study of Tivozanib Eye Drops in Healthy Volunteers and Patients with Neovascular Age-Related Macular Degeneration

**DOI:** 10.1016/j.xops.2024.100553

**Published:** 2024-05-22

**Authors:** Fumi Gomi, Tomohiro Iida, Ryusaburo Mori, Shinya Horita, Hiroaki Nakamura, Yu Nakajima, Ayako Shiokawa, Kanji Takahashi

**Affiliations:** 1 Department of Ophthalmology, Hyogo Medical University, Nishinomiya, Hyogo, Japan; 2 Department of Ophthalmology, Tokyo Women's Medical University, Tokyo, Japan; 3 Division of Ophthalmology, Department of Visual Sciences, Nihon University School of Medicine, Tokyo, Japan; 4 R&D Division, Kyowa Kirin Co., Ltd., Tokyo, Japan; 5 Department of Ophthalmology, Kansai Medical University, Hirakata, Osaka, Japan

**Keywords:** Anti-VEGF, Eye drops, Neovascular age-related macular degeneration, Tivozanib, VEGFR tyrosine kinase inhibitors

## Abstract

**Purpose:**

To evaluate the safety, pharmacokinetics, and exploratory efficacy of tivozanib eye drops in healthy volunteers and patients with neovascular age-related macular degeneration (nAMD).

**Design:**

This multicenter group-sequential dose escalation phase I study consisted of a placebo-controlled double-masked study of healthy volunteers (cohorts 1 and 2) and an open-label study of patients with nAMD (cohort 3).

**Participants:**

Healthy volunteers: Japanese or White men aged 20 to <50 years. Patients with nAMD with central subfield thickness (CST) ≥300 μm and best-corrected visual acuity score ≥23 letters in the study eye.

**Methods:**

In the single-dose cohort of healthy men (cohort 1: steps 1–5), 1 or 2 tivozanib eye drops (30 μL/drop, 5-minute interval; 0.5, 1.0, and 2.0 w/v%) or placebo were administered in 1 eye once. In the multiple-dose cohort of healthy men (cohort 2: steps 1–6), 1 or 2 tivozanib eye drops (0.5, 1.0, and 2.0 w/v%) or placebo were administered 3 times daily in 1 eye for 21 days. In the multiple-dose cohort of patients with nAMD (cohort 3, steps 1–3), 1 or 2 tivozanib eye drops (0.5 and 1.0 w/v%) were administered 3 times daily in 1 affected eye for 21 days.

**Main Outcome Measures:**

The safety outcome measures included adverse events (AEs). The pharmacokinetic outcome was serum tivozanib concentration. Among the exploratory efficacy outcomes, CST was evaluated.

**Results:**

In total, 40, 48, and 28 participants were enrolled in cohorts 1, 2, and 3, respectively. Serious AEs did not occur in cohorts 1 to 3. The most frequent AE in multiple-dose cohorts was reversible punctate keratitis: placebo arm, 8.3% (healthy men, 1/12); tivozanib arm, 47.2% (healthy men, 17/36) and 14.3% (nAMD, 4/28). Serum tivozanib exposure increased dose-dependently and was similar in healthy men and patients with nAMD. In patients with nAMD, mean CST changes from baseline to day 22 were −27.6 ± 54.88 (0.5 w/v%; 1 drop, 3 times daily), −35.6 ± 49.64 (1.0 w/v%; 1 drop, 3 times daily), and −43.7 ± 55.19 μm (1.0 w/v%; 2 drops, 3 times daily).

**Conclusions:**

Tivozanib eye drops showed a favorable safety profile in healthy Japanese and White men and Japanese patients with nAMD.

**Financial Disclosure(s):**

Proprietary or commercial disclosure may be found in the Footnotes and Disclosures at the end of this article.

Age-related macular degeneration is a disease that affects the macular region of the retina, causing progressive central vision loss. The estimated number of people with age-related macular degeneration globally was approximately 200 million by 2020, which is projected to reach 288 million by 2040.^1^

The VEGF pathway plays an important role in the pathology of neovascular age-related macular degeneration (nAMD).^2^ Intravitreal injections of ranibizumab, aflibercept, faricimab, and brolucizumab are currently approved and are the standard of care to treat both nAMD and other retinal diseases.^3^ Although these anti-VEGF biologic agents have shown significant efficacy in patients with nAMD, their delivery to the target tissue requires invasive procedures, frequent (up to monthly) injections, and long-term treatment that involves frequent hospital/clinic visits and surgical interventions. In addition, intravitreal therapy is expensive and not all medical institutions can provide this service. This imposes an increased burden not only on patients and providers but also on caretakers and health care systems.^4^, ^5^, ^6^ Furthermore, intravitreal injections carry the risk of vision-threatening complications, including endophthalmitis and retinal detachment.^7^ To overcome these problems associated with intravitreal treatment, several anti-VEGF eye drops for nAMD have been developed for clinical use, such as pazopanib, regorafenib, and LHA510;^8^, ^9^, ^10^ however, further development of most of these eye drops has been discontinued because of lack of efficacy, and no topical agents have yet been approved for nAMD therapy.

Tivozanib is a tyrosine kinase inhibitor that strongly and selectively inhibits all 3 types of VEGF receptors (VEGFRs) (VEGFR-1, -2, and -3), as well as the proangiogenic platelet-derived growth factor receptor-β.^11^ VEGF plays a central role in angiogenesis and vascular permeability of tumor tissues. By blocking VEGF-induced VEGFR activation, tivozanib inhibits angiogenesis and vascular permeability in tumor tissues, leading to inhibition of tumor growth in vivo.^11^ This antitumor efficacy was also demonstrated in patients with renal cell carcinoma and other solid tumors.^12^

Tivozanib has been developed as an oral formulation and is currently considered a safe and tolerable VEGFR inhibitor for the treatment of renal cell carcinoma.^13^, ^14^, ^15^ The oral formulation containing tivozanib as the active ingredient has been approved by the United States Food and Drug Administration and the European Medicines Agency for the treatment of patients with advanced renal cell carcinoma.^16^^,^^17^

Tivozanib is currently being developed as eye drops to efficiently deliver tivozanib to the target ocular tissues and to exert efficacy while minimizing systemic exposure and adverse drug reactions (e.g., hypertension). It remains to be elucidated if tivozanib eye drops are safe and effective in patients with nAMD.

In this phase I study, the primary objective was to evaluate the safety and tolerability of single or multiple topical ocular doses of tivozanib eye drops in healthy volunteers and patients with nAMD. The other objective was to evaluate the pharmacokinetics of single or multiple topical ocular doses of tivozanib in healthy volunteers and patients with nAMD. In addition, the exploratory efficacy of tivozanib eye drops was evaluated in patients with nAMD.

## Methods

### Study Design

**Figure 1:**
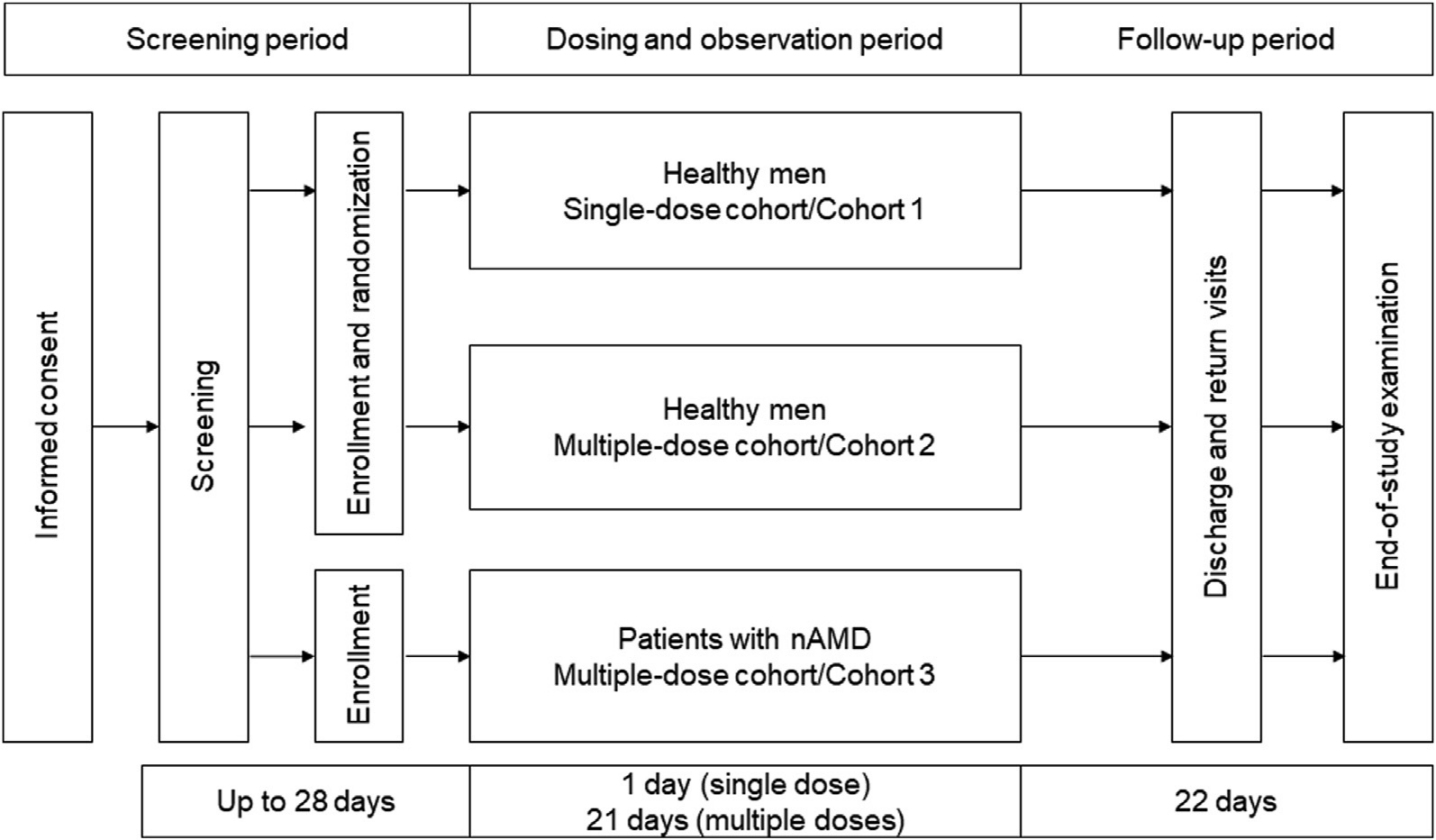
Study design. nAMD = neovascular age-related macular degeneration.

This study comprised a phase I, multicenter (2 sites for healthy volunteers), placebo-controlled, double-masked, group-sequential dose escalation study of healthy men (cohorts 1 and 2) and a phase I, multicenter (14 sites for patients with nAMD), open-label, group-sequential dose escalation study of male and female patients with nAMD (cohort 3). A list of the study sites is included in Table S1 (available at www.ophthalmologyscience.org). The study had a screening period that started at the time of informed consent, a 1-day (single dose) or 21-day (multiple doses) dosing and observation period, and a 22-day follow-up period (Fig 1).

Institutional review board/ethics committee approval was obtained (a list of the institutional review boards is provided in Table S1, available at www.ophthalmologyscience.org). This study was conducted in accordance with the principles that have their origin in the Declaration of Helsinki and in compliance with the Pharmaceuticals and Medical Devices Act, the Ministerial Ordinance on Good Clinical Practice for Drugs (Ordinance of the Ministry of Health and Welfare No. 28 of 27 Mar 1997) and the partial revision of the ordinance, and the International Conference on Harmonisation-E6 Good Clinical Practice (ICH-E6 GCP) guidelines. Written consent was obtained from all patients. This study was registered at ClinicalTrials.gov under the identifier number NCT04594681 and the CONSORT reporting guidelines were referred to during the writing of the manuscript.^18^

### Intervention

**Figure 2:**
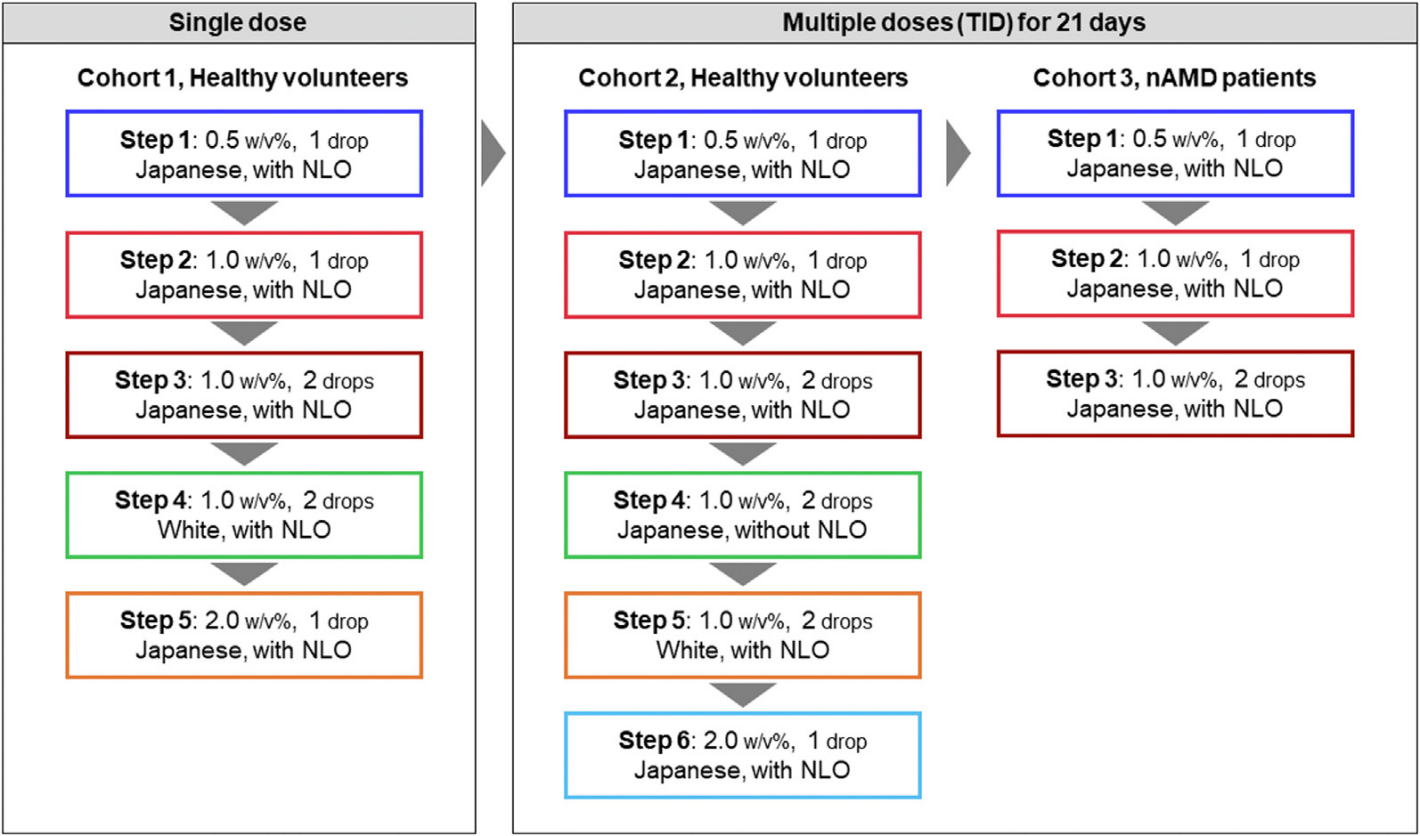
Schematic of study cohorts and steps. nAMD = neovascular age-related macular degeneration; NLO = nasolacrimal duct occlusion or eyelid closure; TID = 3 times daily.

A schematic of the 3 cohorts and steps involved in this phase I study is shown in Figure 2. In the single-dose cohort of healthy men (cohort 1: steps 1–5), tivozanib eye drops or placebo eye drops (that did not contain the active ingredient, tivozanib) were administered (30 μL/drop) once daily (morning) in 1 eye with nasolacrimal duct occlusion or eyelid closure as follows: step 1 (0.15 mg/day group): tivozanib 0.5 percent weight/volume (w/v%) or placebo (Japanese participants; 1 drop), step 2 (0.3 mg/day group): tivozanib 1.0 w/v% or placebo (Japanese participants; 1 drop), step 3 (0.6 mg/day group): tivozanib 1.0 w/v% or placebo (Japanese participants; 2 drops with a 5-minute interval between eye drops), step 4 (0.6 mg/day group): tivozanib 1.0 w/v% or placebo (White participants; 2 drops with a 5-minute interval between eye drops), and step 5 (0.6 mg/day group): tivozanib 2.0 w/v% or placebo (Japanese participants; 1 drop). Participants were hospitalized at the investigative site from 2 days before the start of investigational product administration to the end of the examination on day 3.

In the multiple-dose cohort of healthy men (cohort 2: steps 1–6) tivozanib or placebo was administered 3 times daily (morning, noon, and evening) in 1 eye with nasolacrimal duct occlusion or eyelid closure (in all steps except step 4) for 21 days as follows: step 1 (0.45 mg/day group): tivozanib 0.5 w/v% or placebo (Japanese participants; 1 drop), step 2 (0.9 mg/day group): tivozanib 1.0 w/v% or placebo (Japanese participants; 1 drop), step 3 (1.8 mg/day group): tivozanib 1.0 w/v% or placebo (Japanese participants; 2 drops with a 5-minute interval between eye drops), step 4 (1.8 mg/day group): tivozanib 1.0 w/v% or placebo (Japanese participants; 2 drops with a 5-minute interval between eye drops, without nasolacrimal duct occlusion or eyelid closure), step 5 (1.8 mg/day group): tivozanib 1.0 w/v% or placebo (White participants; 2 drops with a 5-minute interval between eye drops), and step 6 (1.8 mg/day group): tivozanib 2.0 w/v% or placebo (Japanese participants; 1 drop). Participants were hospitalized at the investigative site from 2 days before the start of investigational product administration to the end of the examination on day 23.

In the multiple-dose cohort of patients with nAMD (cohort 3: steps 1 to 4), tivozanib was planned to be administered 3 times daily (morning, noon, and evening) in 1 affected eye with nasolacrimal duct occlusion or eyelid closure (in all steps except step 4) for 21 days as follows: step 1 (0.45 mg/day group): tivozanib 0.5 w/v% (Japanese participants; 1 drop), step 2 (0.9 mg/day group): tivozanib 1.0 w/v% (Japanese participants; 1 drop), step 3 (1.8 mg/day group): tivozanib 1.0 w/v% (Japanese participants; 2 drops with a 5-minute interval between eye drops), and step 4 (1.8 mg/day group): tivozanib 1.0 w/v% (Japanese participants; 2 drops with a 5-minute interval between eye drops, without nasolacrimal duct occlusion or eyelid closure). Participants self-administered the investigational product and recorded the administration status in a patient diary. Of note, during the study conduct, data obtained at the highest dose in cohort 2 step 4 “without nasolacrimal duct occlusion or eyelid closure” indicated no safety concerns, and no new safety concerns were observed in cohort 3 step 2 in patients with nAMD compared with healthy men in a similar setting (cohort 2 step 2). Therefore, the study sponsor determined that the objectives of the study had been satisfied at an earlier stage than expected, and thus, the study was terminated at cohort 3 step 3.

In cohort 3, the use of anti-VEGF intravitreal injections for the study eye was prohibited from 16 weeks before the start of investigational product administration to the end of the observation/examination on day 22. At the time of informed consent, patients agreed to not receive treatment with anti-VEGF injections (the standard of care) in the study eye until the end of the administration phase.

### Participants and Patients

The inclusion criteria in healthy volunteers were as follows: Japanese or White men aged 20 to <50 years at the time of informed consent, body mass index 18.5 to <30.0 kg/m^2^ at screening, intraocular pressure (study eye) 10.0 to 21.0 mmHg, and monocular or binocular visual acuity (corrected visual acuity if correction is required) ≥20/20 at screening. The exclusion criteria in healthy volunteers were the following: current illness requiring treatment; history of ophthalmic laser surgery, ophthalmic surgery, nasolacrimal duct surgery, or any eyelid surgery affecting flow in the nasolacrimal duct; history of or current circulatory disease (e.g., cerebrovascular or cardiovascular disease); history of or current dry eye; and/or abnormal findings on OCT at screening or enrollment examination.

In patients with nAMD, the inclusion criteria were as follows: age ≥50 years at the time of informed consent; nAMD-associated active subfoveal choroidal neovascularization (CNV) lesions in the study eye or juxtafoveal CNV lesions with CNV-associated findings in the fovea of the study eye; central subfield thickness (CST) ≥300 μm in the study eye at screening, as measured by high definition (HD)-OCT; best-corrected visual acuity (BCVA) score ≥23 letters in the study eye at screening and enrollment, as measured by the ETDRS visual acuity chart; BCVA score ≥58 letters in the nonstudy eye at screening, as measured by the ETDRS visual acuity chart; and in patients with prior treatment of the study eye with anti-VEGF drugs such as brolucizumab, aflibercept, ranibizumab, bevacizumab, or pegaptanib sodium, presence of response to the anti-VEGF drug(s) as judged by the investigator or subinvestigator based on OCT, fluorescein angiography, visual acuity, or other assessment results.

In patients with nAMD, the exclusion criteria were as follows: clinical findings or a history of conditions other than nAMD affecting the retina and choroid (e.g., diabetic retinopathy, diabetic macular edema, myopic CNV, retinal vein occlusion, or epiretinal membrane) in either eye; glaucoma, ischemic optic neuropathy, or retinitis pigmentosa in the study eye; current or history of vitreous hemorrhage or macular hole in the study eye; any abnormality in the anterior segment of the eye or vitreous cavity that may affect fundus observation by OCT, color fundus photography, or fluorescein angiography; and/or treatment of the study eye with anti-VEGF drugs within 16 weeks before the start of investigational product administration.

### Randomization and Masking

Participants in the single-dose cohort (cohort 1) and multiple-dose cohort (cohort 2) of healthy men were randomly assigned to receive tivozanib eye drops or placebo by a subject allocation manager according to a computer-generated table of random numbers. All the individuals involved in the study, investigative site staff, and study participants were masked to drug allocation, with the exception of the investigational product manager, the subject allocation manager and personnel, unmasked persons responsible for dosing, unmasked monitors, and the drug concentration measurement facility. Containers of investigational product were packed so that the content was invisible, and an unmasked person responsible for dosing administered the investigational product to the study participants. Masking was maintained by keeping the sponsor, study participants, investigators, and subinvestigators unaware of which investigational product was administered to each participant during the study. All patients with nAMD in the multiple-dose cohort (cohort 3) received tivozanib eye drops in an open-label manner.

### Outcomes

The following safety outcomes were evaluated: treatment-emergent adverse events (TEAEs), laboratory values (hematology, chemistry, coagulation, thyroid function, and urinalysis), vital signs, 12-lead electrocardiogram, body weight, and ophthalmic examinations (visual acuity assessed by the Landolt ring test, intraocular pressure, slit lamp examination, fundus photography, and OCT). A TEAE was defined as any adverse event (AE) that occurred or worsened from the first investigational product administration to the end of the study. Any AE with an outcome of “fatal” was considered an AE leading to death. Serious AEs other than death were considered as other serious AEs. Nonserious AEs leading to withdrawal of the investigational product were considered as other significant AEs. The severity of TEAEs was graded (grades 1–5) according to the National Cancer Institute-Common Terminology Criteria for Adverse Events v5.0.^19^

The pharmacokinetic outcomes were serum tivozanib concentration and pharmacokinetic parameters (including time to reach maximum concentration, maximum concentration [C_max_], area under the concentration–time curve, and elimination half-life). For the measurement of serum tivozanib concentration, blood samples were collected at the following time points: cohort 1, at predose, 0.5, 1, 3, 5, 7, 10, 12, 24, 36, 48, 96, 192, 360, and 528 hours postdose; cohort 2, before the first dose, the second dose, and the third dose on day 1, then, 24, 72, 168, 264, 360, 480, 480.5, 481, 483, 485, 487, 490, 492, 504, 516, 528, 576, 672, 840, and 1008 hours after the first dose on day 1; and cohort 3, on days 1, 8, 15, 22 (1 day after the administration period), and 43 (22 days after the administration period).

The following exploratory efficacy outcomes were evaluated in cohort 3: BCVA as measured by the ETDRS visual acuity chart on screening, enrollment (day 1), and days 8, 22, and 43; CST as assessed by HD-OCT (Carl Zeiss Meditec: Cirrus HD-OCT [model 4000, 400], Cirrus HD-OCT plus [model 5000, 500], and Cirrus HD-OCT [model 6000]; Heidelberg Engineering: SPECTRALIS OCT and SPECTRALIS HRA + OCT; Topcon Healthcare: DRI OCT Triton [Triton plus/Triton]) on screening, enrollment (day 1), and days 8, 22, and 43; retinal morphology and dry macula as assessed by HD-OCT on screening, enrollment (day 1), and days 8, 22, and 43; and total lesion area and total CNV leakage area as measured by fluorescein angiography on screening and day 22. The retinal morphology was assessed by presence or absence of intraretinal fluid (IRF), subretinal fluid (SRF), subretinal pigment epithelium fluid, and subretinal hyperreflective material within a 1-mm diameter circle centered on the fovea on OCT. Dry macula was defined as no IRF or SRF within a 6-mm diameter circle centered on the fovea on OCT. For the evaluation of dry macula, (+) was defined as “Dry” and (−) as “Non-dry.” All imaging scans for exploratory efficacy outcomes were centrally evaluated by the image reading center.

### Statistical Methods

For the single-dose cohort (cohort 1) and the multiple-dose cohort (cohort 2) of healthy men and the multiple-dose cohort of patients with nAMD (cohort 3), the target number of participants in each tivozanib group in each step was 6. The size of the cohort is considered sufficient for the purpose of the study.^20^ The target sample size in the placebo group in each step in cohorts 1 and 2 was set to 2 to avoid assessment bias. Cohort 3 did not include a placebo group because the cohort involved patients and the study in this cohort was designed to be conducted in an open-label manner to ensure safety. No significance level or power was considered when determining the number of participants in each cohort.

The target sample sizes in each cohort were as follows: for the single-dose cohort of healthy men (cohort 1), 8 participants (tivozanib, 6; placebo, 2) per step × 5 steps (40 participants in total); for the multiple-dose cohort of healthy men (cohort 2), 8 participants (tivozanib, 6; placebo, 2) per step × 6 steps (48 participants in total); and for the multiple-dose cohort of patients with nAMD (cohort 3), ≥6 patients (tivozanib, approximately 6–10 patients) per step × 3 steps (approximately 18–30 patients in total).

The modified intention-to-treat set was the primary analysis set for the evaluation of safety and efficacy and included all participants except those who had never been exposed to the investigational product. Pharmacokinetics were evaluated in the pharmacokinetic analysis set, defined as all participants assigned to the tivozanib groups, except those who met either of the following criteria: those who had never received tivozanib as specified in the protocol and those who had never undergone blood sample collection for pharmacokinetic analysis after the administration of tivozanib.

Categorical data were summarized using frequencies and percentages and continuous data were summarized using descriptive statistics (number of participants, mean, standard deviation, minimum, median, and maximum). The treatment compliance rates were calculated as follows: compliance rate (%) = 100 × (actual number of administrations − number of inappropriate dose administrations)/actual number of administrations. The number and percentage of participants with any TEAE were summarized according to the Medical Dictionary for Regulatory Activities, version 25.0, by System Organ Class and preferred term. Drug-related TEAEs were summarized in the same manner as TEAEs.

Regarding the efficacy endpoints, descriptive statistics of measured values and changes from baseline at each time point for each step in cohort 3 were presented for BCVA as measured by the ETDRS visual acuity chart, and CST as measured by HD-OCT. For retinal morphology (IRF, SRF, subretinal pigment epithelium fluid, and subretinal hyperreflective material) and dry macula as assessed by HD-OCT, the participants with morphological changes at each time point were presented for each step in cohort 3.

Missing data were handled as missing values and were not imputed by any statistical procedure. All statistical analyses were performed using SAS Life Science Analytics Framework 5.2 and 5.4 (SAS 9.4; SAS Institute Inc).

## Results

### Study Participants and Patients

**Figure 3:**
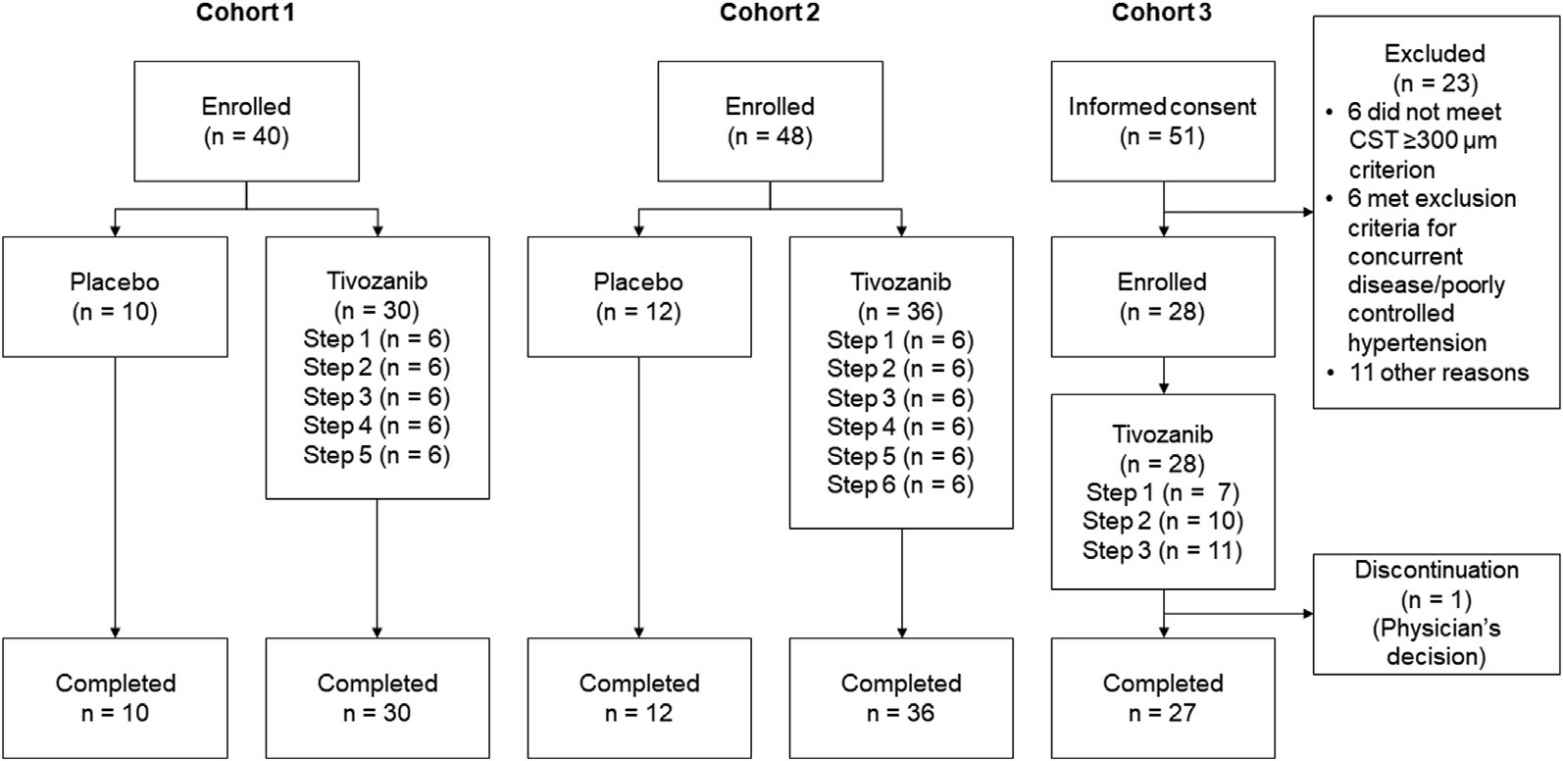
Patient disposition. CST = central subfield thickness.

The disposition of participants and patients is shown in Figure 3. In total, 40 and 48 participants were enrolled and received ≥1 dose of the investigational product in cohorts 1 and 2, respectively, and 51 patients provided informed consent in cohort 3. In cohorts 1 and 2, all participants completed the study. In cohort 3, 23 patients were excluded (with the most common reasons being not meeting inclusion criteria such as CST ≥300 μm in the study eye at screening, as measured by HD-OCT, n = 6; concurrent disease/poorly controlled hypertension, n = 6; and other reasons, n = 11); thus, 28 of the 51 patients who were eligible were enrolled and received tivozanib. Among them, 27 patients completed the study. The modified intention-to-treat set comprised 40 participants in cohort 1 (steps 1–5: 6 participants each received tivozanib, 10 participants received placebo), 48 participants in cohort 2 (steps 1–6: 6 participants each received tivozanib, 12 participants received placebo), and 28 patients in cohort 3 (step 1: 7 patients, step 2: 10 patients, step 3: 11 patients).

Baseline demographic and ocular characteristics in cohorts 1, 2, and 3 are summarized in Tables 1 and 2. Except for weight and/or height in White participants (i.e., cohort 1 step 4 and cohort 2 step 5), there were no notable differences in the background characteristics in cohorts 1 and 2 among the tivozanib groups in all steps and the placebo group. Similarly, there were no notable differences in the background characteristics of patients in cohort 3 among the steps. In cohort 3, 22 of 28 patients (78.6%) were treatment-naive and 6 patients (21.4%) had been previously treated with anti-VEGF injections (Table 2).

**Table 1:** Baseline Demographic Characteristics of Heathy Volunteers in Cohorts 1 and 2

Characteristics	Cohort 1		Cohort 2	
	Tivozanib (n = 30)	Placebo (n = 10)	Tivozanib (n = 36)	Placebo (n = 12)
Sex, male	30 (100.0)	10 (100.0)	36 (100.0)	12 (100.0)
Race				
Japanese	24 (80.0)	8 (80.0)	30 (83.3)	10 (83.3)
White	6 (20.0)	2 (20.0)	6 (16.7)	2 (16.7)
Age, yrs	29.9 ± 8.7	27.5 ± 8.7	28.8 ± 6.0	27.6 ± 6.4
Weight, kg	67.8 ± 9.6	67.5 ± 10.0	66.0 ± 7.2	67.3 ± 11.5
Height, cm	172.0 ± 6.6	173.1 ± 6.8	174.0 ± 6.4	173.9 ± 7.9
BMI, kg/m^2^	22.9 ± 2.8	22.5 ± 2.8	21.8 ± 2.2	22.1 ± 2.5
Study eye, left	29 (96.7)	10 (100.0)	36 (100.0)	11 (91.7)

BMI = body mass index; SD = standard deviation.

Data are shown as n (%) or mean ± SD.

**Table 2:** Baseline Demographic and Ocular Characteristics of Patients with nAMD in Cohort 3

Characteristics	Cohort 3
	Tivozanib (n = 28)
Sex, male	19 (67.9)
Race	
Japanese	28 (100.0)
White	N/A
Age, yrs	71.0 ± 5.8
Weight, kg	64.1 ± 10.0
Height, cm	161.4 ± 6.7
BMI, kg/m^2^	24.6 ± 3.5
Study eye, left	14 (50.0)
Duration of nAMD, years	0. 9 ± 2.1
Subtype of CNV lesion	
Predominantly classic CNV	3 (10.7)
Minimally classic CNV	6 (21.4)
Occult with no classic CNV	19 (67.9)
Type of nAMD	
Typical AMD	16 (57.1)
PCV	12 (42.9)
Drusen in study eye, with	17 (60.7)
Previous use of anti-VEGF treatment, yes	6 (21.4)
Smoker	7 (25.0)

BMI = body mass index; CNV = choroidal neovascularization; nAMD = neovascular age-related macular degeneration; PCV = polypoidal choroidal vasculopathy; SD = standard deviation.

Data are shown as n (%) or mean ± SD.

In cohorts 1 and 2, the treatment compliance rates were 100% because all participants were hospitalized and administered the investigational product by site staff. In cohort 3, the mean treatment compliance rates were 100% in step 1, 99.8% in step 2%, and 99.5% in step 3. In cohort 3, administration status was self-reported using patient diaries at every visit.

### Safety

No deaths, other serious AEs, or other significant AEs occurred in cohort 1 (Table S2, available at www.ophthalmologyscience.org). Adverse events and ocular AEs in the study eye in cohort 2 are summarized in Table 3 and AEs other than ocular AEs in cohort 2 are shown in Table S3 (available at www.ophthalmologyscience.org). In the tivozanib group of cohort 2, any TEAE occurred in 28 (77.8%) participants and drug-related TEAEs occurred in 16 (44.4%) participants. No deaths, other serious AEs, or other significant AEs occurred in cohort 2. In cohort 2, the most frequently reported TEAE was punctate keratitis (placebo: n = 1 [8.3%]; steps 1 and 2: n = 2 [33.3%] each; step 3: n = 1 [16.7%]; step 4: n = 6 [100%]; steps 5 and 6: n = 3 [50.0%] each) followed by eye irritation (step 2: n = 3 [50.0%]; step 3: n = 1 [16.7%]; step 4: n = 4 [66.7%]; step 5: n = 1 [16.7%]; step 6: n = 3 [50.0%]). Individual participant visual acuity assessed by the Landolt ring test for healthy volunteers in cohorts 1 and 2 are shown in Tables S4 and S5 (available at www.ophthalmologyscience.org). At the same dosing regimen, the incidence of TEAEs between tivozanib 1.0 w/v% (step 3) and tivozanib 2.0 w/v% (step 6) formulations was comparable. By reviewing the results of Japanese (step 3) and White (step 5) participants with the same dosing regimen, the overall incidence of TEAEs in Japanese participants was slightly higher than that in White participants. However, the safety profile between Japanese and White participants was similar.

**Table 3:** Summary of Adverse Events and Ocular Adverse Events in the Study Eye in Cohort 2

Adverse Events	Placebo n = 12		Tivozanib													
			Step 1 0.45 mg/day Japanese with∗ 0.5 w/v% (TID) n = 6		Step 2 0.9 mg/day Japanese with∗ 1.0 w/v% (TID) n = 6		Step 3 1.8 mg/day Japanese with∗ 1.0 w/v% (TID) n = 6		Step 4 1.8 mg/day Japanese without† 1.0 w/v% (TID) n = 6		Step 5 1.8 mg/day White with∗ 1.0 w/v% (TID) n = 6		Step 6 1.8 mg/day Japanese with∗ 2.0 w/v% (TID) n = 6		Total n = 36	
	n	(%)	n	(%)	n	(%)	n	(%)	n	(%)	n	(%)	n	(%)	n	(%)
Any TEAE	2	(16.7)	3	(50.0)	4	(66.7)	6	(100.0)	6	(100.0)	4	(66.7)	5	(83.3)	28	(77.8)
Death	0		0		0		0		0		0		0		0	
Other serious	0		0		0		0		0		0		0		0	
Other significant	0		0		0		0		0		0		0		0	

Any drug-related TEAE	0		0		3	(50.0)	2	(33.3)	6	(100.0)	2	(33.3)	3	(50.0)	16	(44.4)
Death	0		0		0		0		0		0		0		0	
Other serious	0		0		0		0		0		0		0		0	
Other significant	0		0		0		0		0		0		0		0	

Any TEAE of the study eye	1	(8.3)	2	(33.3)	4	(66.7)	2	(33.3)	6	(100.0)	3	(50.0)	5	(83.3)	22	(61.1)
Any drug-related TEAE of the study eye	0		0		3	(50.0)	1	(16.7)	6	(100.0)	2	(33.3)	3	(50.0)	15	(41.7)

Ocular adverse events in the study eye																
Eye disorders	1	(8.3)	2	(33.3)	4	(66.7)	2	(33.3)	6	(100.0)	3	(50.0)	5	(83.3)	22	(61.1)
Punctate keratitis	1	(8.3)	2	(33.3)	2	(33.3)	1	(16.7)	6	(100.0)	3	(50.0)	3	(50.0)	17	(47.2)
Eye irritation	0		0		3	(50.0)	1	(16.7)	4	(66.7)	1	(16.7)	3	(50.0)	12	(33.3)
Foreign body sensation in eyes	0		0		0		0		1	(16.7)	0		1	(16.7)	2	(5.6)
Vision blurred	0		0		0		0		1	(16.7)	0		0		1	(2.8)
Eye pruritus	0		0		0		0		0		1	(16.7)	0		1	(2.8)

TEAE = treatment-emergent adverse event; TID = 3 times daily.

∗ With nasolacrimal duct occlusion or eyelid closure.

† Without nasolacrimal duct occlusion or eyelid closure.

Adverse events and ocular AEs in the study eye in cohort 3 are shown in Table 4 and AEs other than ocular AEs in cohort 3 are shown in Table S6 (available at www.ophthalmologyscience.org). In cohort 3, any TEAE occurred in 12 (42.9%) participants, and drug-related TEAEs occurred in 7 (25.0%) participants among those treated with tivozanib. No deaths, other serious AEs, or other significant AEs occurred in cohort 3. In cohort 3, the most frequently reported TEAE was punctate keratitis (step 2: n = 1 [10.0%]; step 3: n = 3 [27.3%]), followed by eye irritation (step 2: n = 2 [20.0%]). Regarding the severity of punctate keratitis observed in the study eye of 21 participants who were administered tivozanib eye drops, 20 were grade 1 (asymptomatic or mild symptoms; intervention not indicated), and 1 was grade 2 (moderate; local or noninvasive intervention indicated).

**Table 4:** Summary of Adverse Events and Ocular Adverse Events in the Study Eye in Cohort 3

Adverse Events	Tivozanib							
	Step 1 0.45 mg/day Japanese with∗ 0.5 w/v% (TID) n = 7		Step 2 0.9 mg/day Japanese with∗ 1.0 w/v% (TID) n = 10		Step 3 1.8 mg/day Japanese with∗ 1.0 w/v% (TID) n = 11		Total n = 28	
	n	(%)	n	(%)	n	(%)	n	(%)
Any TEAE	3	(42.9)	4	(40.0)	5	(45.5)	12	(42.9)
Death	0		0		0		0	
Other serious	0		0		0		0	
Other significant	0		0		0		0	

Any drug-related TEAE	1	(14.3)	2	(20.0)	4	(36.4)	7	(25.0)
Death	0		0		0		0	
Other serious	0		0		0		0	
Other significant	0		0		0		0	

Any TEAE of the study eye	1	(14.3)	4	(40.0)	3	(27.3)	8	(28.6)
Any drug-related TEAE of the study eye	1	(14.3)	2	(20.0)	3	(27.3)	6	(21.4)

Ocular adverse events in the study eye								
Eye disorders	1	(14.3)	4	(40.0)	3	(27.3)	8	(28.6)
Punctate keratitis	0		1	(10.0)	3	(27.3)	4	(14.3)
Eye irritation	0		2	(20.0)	0		2	(7.1)
Eye pain	0		1	(10.0)	0		1	(3.6)
Lacrimation increased	0		1	(10.0)	0		1	(3.6)
Pinguecula	1	(14.3)	0		0		1	(3.6)
Swelling of eyelid	0		1	(10.0)	0		1	(3.6)

TEAE = treatment-emergent adverse event; TID = 3 times daily.

∗ With nasolacrimal duct occlusion or eyelid closure.

No grade ≥3 TEAEs occurred in this study. No significant safety concerns were identified for laboratory values, vital signs, 12-lead electrocardiogram, body weight, or ophthalmic examinations in any cohorts.

### Pharmacokinetics

**Figure 4:**
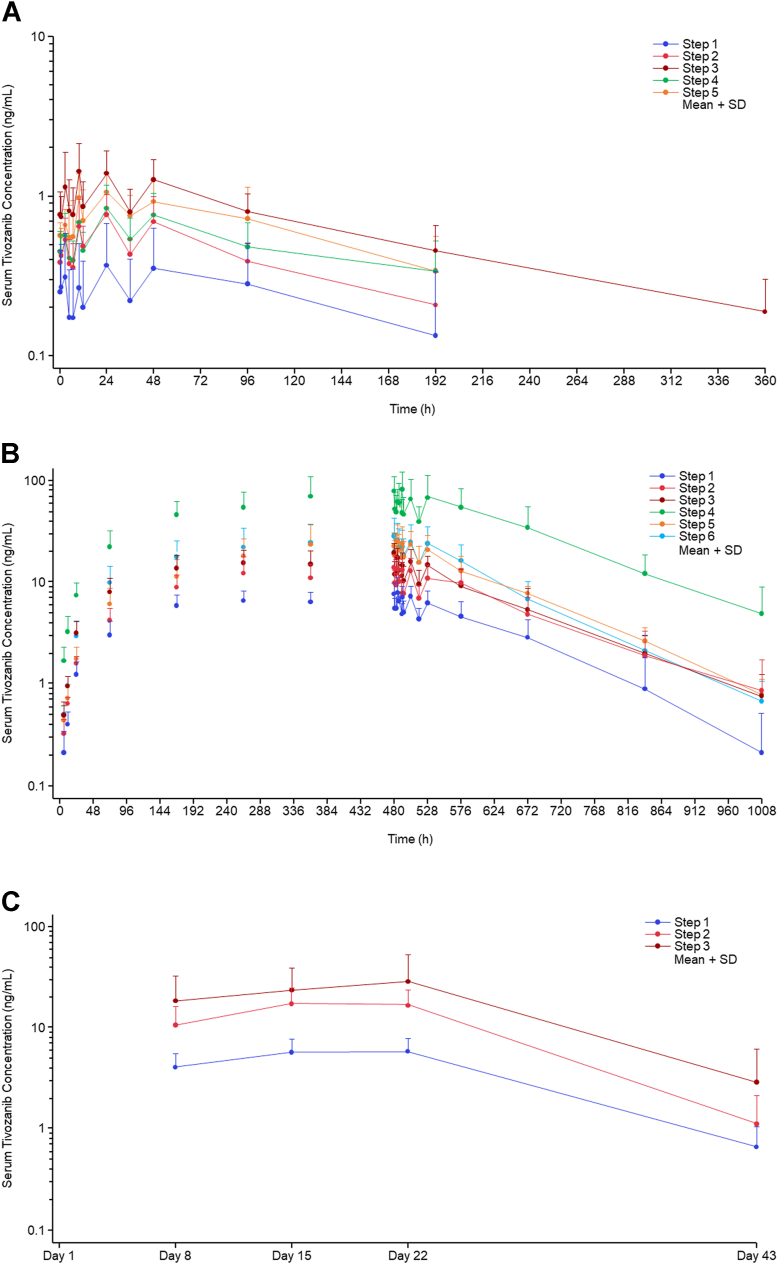
Serum tivozanib concentration–time profiles in (**A**) cohort 1, (**B**) cohort 2, and (**C**) cohort 3^∗^. Serum tivozanib concentrations at 528 hours were below the lower limit of quantification in all steps of cohort 1. ^∗^Intravitreal injection of anti-VEGF drugs was allowed after the end of the eye drop administration phase (after the end of day 22). SD = standard deviation.

In cohort 1, after a single dose of tivozanib in healthy Japanese men, the mean serum tivozanib concentration increased in a dose-dependent manner (Fig 4A; step 1, step 2, and step 3, including step 5), and C_max_, area under the concentration–time curve from time zero to the last measurable time point (AUC_0–t_), and area under the concentration–time curve from time zero to infinity (AUC_0–∞_) increased with dose. In cohort 2, after the multiple doses of tivozanib in healthy Japanese men, the mean serum tivozanib concentration increased in a dose-dependent manner (Fig 4B; step 1, step 2, and step 3, including step 6), and C_max_, AUC_0–t_, and AUC_0–∞_ increased with dose. In the comparison between single and multiple administration, there seemed to be no notable differences in the pharmacokinetics of tivozanib, except for an increase in exposure due to multiple dosing (Fig 4A, B and Table 5). In cohort 2, after multiple doses of tivozanib, the mean serum tivozanib trough concentrations seemed to be almost stable around 360 hours after the first administration of tivozanib, and thus the concentrations were considered to have reached a steady state around that time in all the steps (Fig 4B).

**Table 5:** Summary of Pharmacokinetic Parameters in Japanese Participants in Cohorts 1 and 2 (Pharmacokinetic Analysis Set)

Step	Treatment Group	t_max_ (hrs)	C_max_ (ng/mL)	AUC_0–t_ (ng·h/mL)	AUC_0–∞_ (ng·h/mL)	AUC_0–τ_ (ng·h/mL)	t_1/2_ (hrs)
Cohort 1							
1	0.15 mg/day Japanese with∗ 0.5 w/v% (N = 6)	24.00 (3.00–48.00)	0.389 ± 0.300	54.8 ± 68.5	151 ± 109	-	123 ± 28
2	0.3 mg/day Japanese with∗ 1.0 w/v% (N = 6)	24.00 (3.00–48.00)	0.790 ± 0.264	90.1 ± 33.1	123 ± 29	-	130 ± 38
3	0.6 mg/day Japanese with∗ 1.0 w/v% (N = 6)	17.00 (10.00–24.00)	1.59 ± 0.60	225 ± 93	306 ± 73	-	124 ± 18
4	0.6 mg/day White with∗ 1.0 w/v% (N = 6)	24.00 (10.00–24.00)	0.852 ± 0.315	120 ± 64	138 ± 63	-	104 ± 28
5	0.6 mg/day Japanese with∗ 2.0 w/v% (N = 6)	24.00 (10.00–48.00)	1.17 ± 0.32	166 ± 105	191 ± 118	-	94.6 ± 25.3
Cohort 2							
1	0.45 mg/day (TID) Japanese with∗ 0.5 w/v% (N = 6)	6.50 (3.00–24.00)	8.48 ± 3.04	1320 ± 572	1370 ± 611	50.9 ± 14.2	89.2 ± 29.2
2	0.9 mg/day (TID) Japanese with∗ 1.0 w/v% (N = 6)	10.00 (3.00–24.00)	13.9 ± 4.0	2470 ± 1057	2700 ± 1243	83.2 ± 25.7	125 ± 69
3	1.8 mg/day (TID) Japanese with∗ 1.0 w/v% (N = 6)	3.00 (3.00–24.00)	17.8 ± 4.9	2760 ± 1123	2890 ± 1190	111 ± 33	117 ± 29
4	1.8 mg/day (TID) Japanese without† 1.0 w/v% (N = 6)	10.00 (10.00–10.00)	83.5 ± 39.4	15 600 ± 8365	16 500 ± 9137	463 ± 194	112 ± 14
5	1.8 mg/day (TID) White with∗ 1.0 w/v% (N = 6)	6.50 (3.00–24.00)	26.4 ± 10.7	3950 ± 1196	4070 ± 1251	167 ± 73	103 ± 10
6	1.8 mg/day (TID) Japanese with∗ 2.0 w/v% (N = 6)	4.00 (3.00–24.00)	27.4 ± 11.9	4110 ± 1815	4200 ± 1864	177 ± 84	98.2 ± 9.9

AUC_0–t_ = area under the concentration–time curve from time zero to the last measurable time point; AUC_0–τ_ = area under the concentration–time curve during dose interval; AUC_0–∞_ = area under the concentration–time curve from time zero to infinity; C_max_ = maximum concentration; SD = standard deviation; TID = 3 times daily; t_max_ = time to reach maximum concentration; t_1/2_ = elimination half-life.

Data are shown as mean ± SD or median (range).

∗ With nasolacrimal duct occlusion or eyelid closure.

† Without nasolacrimal duct occlusion or eyelid closure.

In cohort 1, after the single dose of tivozanib in healthy Japanese and White men, the mean serum tivozanib concentration over time was greater in Japanese participants (step 3) compared with that in White participants (step 4) (Fig 4A). In cohort 2, after the multiple doses of tivozanib in healthy Japanese and White men, the mean serum tivozanib concentration was greater in Japanese participants (step 3) compared with White participants (step 5) following tivozanib administration, whereas from 264 hours after the first administration of tivozanib, the mean serum tivozanib concentration was lower in Japanese participants compared with White participants (Fig 4B).

In cohort 1, following single administration of tivozanib 1.0 w/v% (step 3) or tivozanib 2.0 w/v% (step 5) with the same dosing regimen in healthy Japanese men, the mean exposures (C_max_, AUC_0–t_, and AUC_0–∞_) with tivozanib 1.0 w/v% (step 3) were approximately 36% to 60% greater than those with tivozanib 2.0 w/v% (step 5) (Fig 4A, Table 5). In cohort 2, following multiple administration of tivozanib 1.0 w/v% (step 3) or tivozanib 2.0 w/v% (step 6) with the same dosing regimen in healthy Japanese men, the mean exposures (C_max_, AUC_0–t_, AUC_0–∞_, and area under the concentration–time curve during dose interval [AUC_0–τ_]) with tivozanib 2.0 w/v% (step 6) were approximately 45% to 59% greater than those with tivozanib 1.0 w/v% (step 3) (Fig 4B, Table 5).

When comparing pharmacokinetics between tivozanib administration “with nasolacrimal duct occlusion or eyelid closure” (step 3) and “without nasolacrimal duct occlusion or eyelid closure” (step 4) at the same dose level following multiple administration, the mean serum tivozanib concentration over time was greater in step 4 compared with that in step 3 (Fig 4B). The mean ± standard deviation C_max_ after the last administration of tivozanib on day 21 was 17.8 ± 4.9 ng/mL in step 3 and 83.5 ± 39.4 ng/mL in step 4 (Table 5). The AUC_0–τ_ was 111 ± 33 ng·h/mL in step 3 and 463 ± 194 ng·h/mL in step 4. The mean C_max_ and AUC_0–τ_ in step 4 were approximately 4.7 times and 4.2 times greater than those in step 3, respectively.

In cohort 3, after the multiple doses of tivozanib in patients with nAMD, the mean serum tivozanib concentrations increased in a dose-dependent manner (Fig 4C). Although the mean serum tivozanib trough concentrations in step 3 after day 15 were still increasing slightly, the concentrations were almost stable in steps 1 and 2; thus, the concentrations were considered to have reached a steady state around day 15 (Fig 4C). Although the serum tivozanib concentrations in Japanese patients with nAMD in steps 2 and 3 were greater than those in healthy Japanese men, there seemed to be no notable differences in the concentrations between healthy Japanese men and Japanese patients with nAMD despite the differences in baseline demographic characteristics (Table S7, available at www.ophthalmologyscience.org).

### Exploratory Efficacy Outcomes

**Figure 5:**
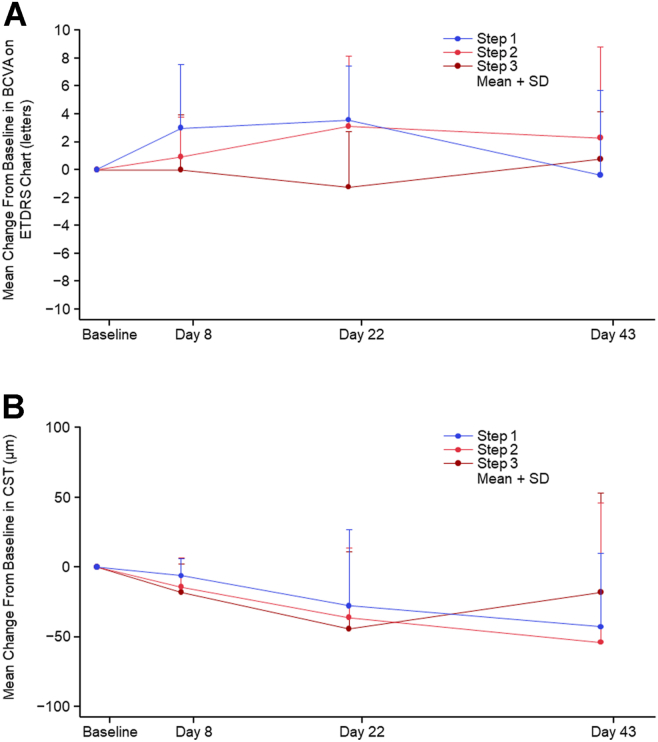
Mean change from baseline in (**A**) BCVA assessed by ETDRS chart and (**B**) CST assessed by OCT in cohort 3 (mITT set). Intravitreal injection of anti-VEGF drugs was allowed after the end of the eye drop administration phase (after the end of day 22). BCVA = best-corrected visual acuity; CST = central subfield thickness; mITT = modified intent-to-treat; SD = standard deviation.

In cohort 3, no patient had a decrease in BCVA of ≥10 letters (2 lines) from baseline to day 22, and most patients maintained BCVA. The mean BCVA at baseline was 61.6 ± 16.17 letters, 75.2 ± 13.66 letters, and 76.2 ± 10.59 letters in steps 1, 2, and 3, respectively. The mean change in BCVA from baseline to day 22 was 3.6 ± 3.87 letters, 3.1 ± 5.00 letters, and −1.3 ± 4.03 letters in steps 1, 2, and 3, respectively (Fig 5A). The BCVA change from baseline to day 22 was >5 letters in 5 patients, between −5 and 5 letters in 19 patients, and <−5 letters in 4 patients. Individual patient data on BCVA as measured by the ETDRS visual acuity chart for patients with nAMD in cohort 3 are shown in Table S8 (available at www.ophthalmologyscience.org).

The mean CST at baseline was 412.9 ± 161.18 μm, 410.7 ± 103.28 μm, and 413.3 ± 230.01 μm in steps 1, 2, and 3, respectively, and at day 22, 385.3 ± 119.89 μm, 375.1 ± 92.25 μm, and 369.5 ± 195.47 μm in each step, respectively. The mean change from baseline to day 22 in CST was −27.6 ± 54.88 μm, −35.6 ± 49.64 μm, and −43.7 ± 55.19 μm in steps 1, 2, and 3, respectively (Fig 5B). Of note, the intravitreal injection of anti-VEGF drugs was not administered until the end of the administration phase (at the end of the day 22 examination and observation).

Significant anatomical changes assessed by OCT were observed in 12 patients from cohort 3 (Table 6). Intraretinal fluid disappeared in 1 patient on day 22. Subretinal fluid disappeared in 1 patient on day 8 and in 4 patients on day 22. One patient had dry macula on day 8, and 3 patients had dry macula on day 22; thus, the rate of dry macula was 14.3% on day 22. Subretinal hyperreflective material appeared in 1 patient on day 8. Of note, patients F and K were administrated intravitreal injection of anti-VEGF drugs at the investigator’s discretion between the end of day 22 and day 43.

**Table 6:** Summary of Anatomical Changes on OCT in Cohort 3

Step	Participant ID	Enrollment, Day 1 (Baseline)	Day 8	Day 22 (after 3-wk Administration Period)	Day 43 (after the Follow-Up Period)
Step 1 (n = 7)	Patient A	IRF (−), SRF (−), sub-RPE fluid (+), SHRM (+)			IRF (+), SRF (+)
	Patient B	IRF (−), SRF (+), sub-RPE fluid (−), SHRM (−)	SHRM (+)	SHRM (+)	SHRM (+)
	Patient C	IRF (−), SRF (+), sub-RPE fluid (−), SHRM (−)		Dry macula (+)	Dry macula (+)

Step 2 (n = 10)	Patient D	IRF (+), SRF (+), sub-RPE fluid (+), SHRM (+)		IRF (−)	IRF (−), sub-RPE fluid (−)
	Patient E	IRF (−), SRF (+), sub-RPE fluid (−), SHRM (−)	Dry macula (+)	Dry macula (+)	Dry macula (+)
	Patient F	IRF (−), SRF (+), sub-RPE fluid (+), SHRM (−)		Dry macula (+)	Dry macula (+), sub-RPE fluid (−)∗
	Patient G	IRF (+), SRF (+), sub-RPE fluid (−), SHRM (+)			IRF (−), SHRM (−)
	Patient H	IRF (−), SRF (+), sub-RPE fluid (+), SHRM (−)			Dry macula (+)

Step 3 (n = 11)	Patient I	IRF (−), SRF (+), sub-RPE fluid (−), SHRM (−)		Dry macula (+)	Dry macula (+)
	Patient J	IRF (−), SRF (+), sub-RPE fluid (+), SHRM (−)		SRF (−)	
	Patient K	IRF (−), SRF (+), sub-RPE fluid (−), SHRM (−)			SRF (−)∗
	Patient L	IRF (−), SRF (+), sub-RPE fluid (−), SHRM (−)			Dry macula (+)

ID = identification; IRF = intraretinal fluid; RPE = retinal pigment epithelium; SHRM = subretinal hyperreflective material; SRF = subretinal fluid.

No significant anatomical change was observed in patients who have not been listed in this table.

For SRF, IRF, sub-RPE fluid, and SHRM, (−) means that these fluids and SHRM disappeared.

For dry macula, (+) means that the macula became dry.

If anatomical changes were observed on OCT compared with the enrollment visit (day 1), those changes are shown in this table on day 8, 22, and 43 (blank spaces mean that no change was observed compared with the enrollment visit [day 1]).

∗ After administering intravitreal injection of anti-VEGF drugs between the end of day 22 and day 43.

**Figure 6:**
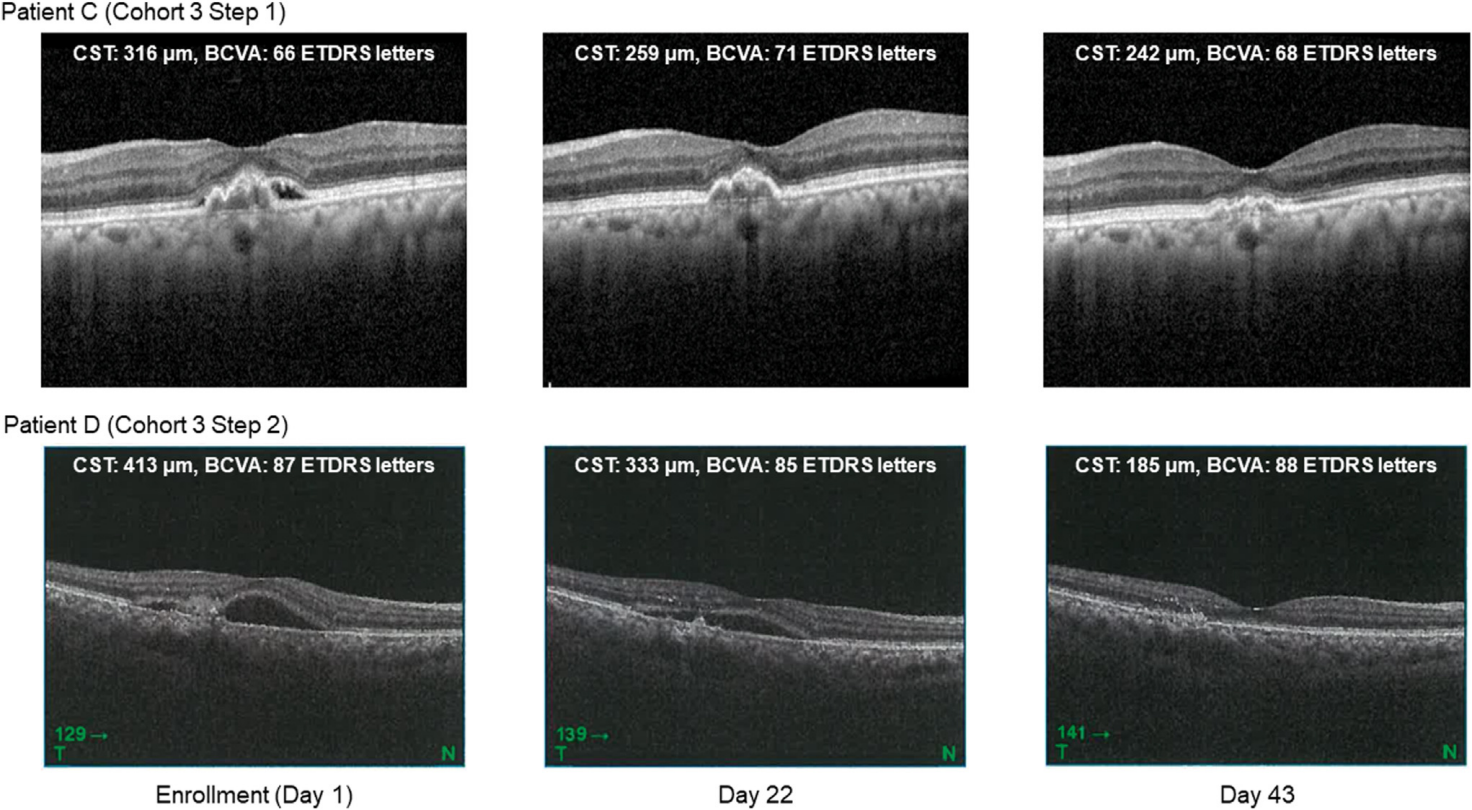
Horizontal OCT scans through the central fovea of 2 patients who showed improvements on OCT. BCVA = best-corrected visual acuity; CST = central subfield thickness.

Figure 6 shows the OCT scans of 2 patients (patients C and D from Table 6) who showed improvements on OCT. In both patients, a significant decrease in CST from preexamination to enrollment (spontaneous remission) was not observed (patient C: +2 μm, patient D: −3 μm). In patient C, disappearance of SRF was observed until day 22, resulting in dry macula. In patient D, disappearance of IRF and reduction of SRF volume were observed by day 22. Resolution of IRF in patient D was noted by the image reading center from the OCT images taken on day 22. In both patients, CST decreased markedly from baseline (enrollment). No increase in the total lesion area or total CNV leakage area was observed from screening to day 22 (after the end of administration). The total lesion area changed from 2.89 mm^2^ to 2.48 mm^2^ in patient C; it remained at 1.80 mm^2^ in patient D. The total CNV leakage area changed from 2.01 mm^2^ to 1.99 mm^2^ in patient C and from 0.26 mm^2^ to 0.13 mm^2^ in patient D.

## Discussion

The present study aimed to evaluate the safety, tolerability, and pharmacokinetics of single or multiple doses of tivozanib eye drops in healthy volunteers and patients with nAMD. In this phase I study, no significant safety concerns were raised in relation to treatment with tivozanib eye drops within the administered dosing regimens in Japanese/White healthy volunteers or Japanese patients with nAMD, although the mean serum tivozanib concentrations increased in a dose-dependent manner. Regarding exploratory efficacy data from patients with nAMD, the results revealed that some patients showed positive signs after a short period of administration (3 times daily for 3 weeks) with a noninvasive method (eye drops), such as the decrease in mean CST from baseline to day 22 and increase in BCVA by >5 letters from baseline at day 22 in 5 patients.

In the present study, there were no serious AEs or AEs leading to discontinuation, and tivozanib showed good tolerability in healthy male Japanese and White volunteers and male and female Japanese patients with nAMD without any significant safety concerns. Superficial punctate keratitis was observed in the study eye of 21 participants who were administered tivozanib eye drops (cohort 1: 0; cohort 2: 17; cohort 3: 4), but all cases except for 1 were assessed as grade 1 and resolved without any treatment; 6 participants recovered during the administration period. Eye irritation was observed in the study eye of 21 participants who were administered tivozanib eye drops (cohort 1: 7; cohort 2: 12; cohort 3: 2), but all recovered within 30 seconds to 30 minutes after drop administration. In previous clinical trials of oral tivozanib, the most common AE was hypertension, which occurred in approximately 50% of patients;^17^ however, in this study, there was 1 case of an increase in blood pressure in cohort 1 step 2 (single-dose cohort), but no hypertension-related AEs in cohorts 2 or 3 (multiple-dose cohorts).

In the present study, systemic tivozanib exposure was higher according to the dose increase and open-eyelid administration. However, the exposure remained at the same level or below that of patients who received tivozanib 1.5 mg/day by oral administration for 3 weeks.^12^ Compared with oral administration, minimization of systemic exposure and fewer adverse drug reactions are considered advantages of eye drops.

Nasolacrimal duct occlusion and eyelid closure are techniques used to increase the ocular bioavailability of topical ocular drugs and decrease adverse effects. A previous study of 0.5% timolol maleate showed that systemic drug absorption after administration of the study drug significantly decreased by using nasolacrimal duct occlusion and eyelid closure.^21^ Similarly, in the present study, nasolacrimal duct occlusion (digital pressure over the lacrimal sac on the side of the study eye for 1 minute) and eyelid closure immediately after eye drop application inhibited the serum transfer of the drug. A possible reason why lacrimal sac compression and eyelid closure can decrease systemic exposure is that a part of the ophthalmic solution overflows from the ocular surface by the procedures. Therefore, nasolacrimal duct occlusion and eyelid closure after eye drop application is important to minimize the occurrence of systemic adverse effects.

Regarding the comparison between Japanese and White participants, the mean serum tivozanib concentration just after starting tivozanib treatment was higher in Japanese participants compared with White participants in both cohorts 1 and 2; however, after multiple administration of tivozanib, the serum tivozanib concentration was lower in Japanese participants compared with White participants in cohort 2. In previous clinical trials of oral tivozanib, the serum drug concentrations were higher in Japanese participants compared with White patients.^12^^,^^22^ The results of racial differences in single-dose and multiple-dose exposure are controversial, and in this phase I study, we could not conclude that exposure was higher for either participant group.

As for the exploratory efficacy, improvements in BCVA, CST, and retinal morphology were observed in some patients. Moreover, mean changes in the total lesion area and total CNV leakage area were stable from screening to day 22. Although further study is needed, based on the exploratory efficacy results, it might be possible that the noninvasive method of administration, using eye drops, could have an effect on the lesion site, although this warrants additional investigation.

The present study has some limitations. The sample size was small, and the administration period was short (3 weeks). During the follow-up period (from day 22 onward), a few participants received intravitreal injections of anti-VEGF agents in all steps, which affected the exploratory efficacy outcomes on day 43. The present study was not designed to evaluate efficacy as a primary endpoint, and further studies are warranted to confirm the efficacy of tivozanib eye drops.

In conclusion, the present study showed that tivozanib eye drop use for 3 weeks was safe and well tolerated. In addition, regarding exploratory efficacy, positive clinical signs were observed in some Japanese patients with nAMD, although further studies are needed to confirm the efficacy of tivozanib eye drops.

## Data Availability

The datasets generated and/or analyzed will be available in the Vivli repository, https://vivli.org/ourmember/kyowa-kirin/, as long as conditions of data disclosure specified in the policy section of the Vivli website are satisfied.
