# Supplementary material for: Phase I Study of Tivozanib Eye Drops in Healthy Volunteers and Patients with Neovascular Age-Related Macular Degeneration
Source: Ophthalmol Sci. 2024 May 22;4(6):100553. doi: 10.1016/j.xops.2024.100553 (PMC11331923; doi:10.1016/j.xops.2024.100553)
Supplement: Supplemental Table 1 [file mmc1.pdf]

Table S1. List of Study Sites and Institutional Review Boards

Site No.	Site Name	IRB Name	Site Address
1	Souseikai Hakata Clinic	Hakata Clinic Institutional Review Board	6-18 Tenyamachi, Hakata-ku, Fukuoka-shi, Fukuoka 812-0025, Japan
2	Kansai Medical University Hospital	Kansai Medical University Hospital Institutional Review Board	2-3-1 Shinmachi, Hirakata-shi, Osaka 573-1191, Japan
3	Nagoya City University Hospital	Nagoya City University Institutional Review Board	1 Kawasumi, Mizuho-cho, Mizuho-ku, Nagoya-shi, Aichi 467-8602, Japan
4	Nihon University Hospital	Nihon University Hospitals' Joint Institutional Review Board	1-6 Surugadai, Kanda, Chiyoda-ku, Tokyo 101-8309, Japan
5	Osaka Metropolitan University Hospital	Osaka Metropolitan University Hospital Clinical Trial Institutional Review Board	1-5-7 Asahi-machi, Abeno-ku, Osaka-shi, Osaka, 545-8586, Japan
6	Hayashi Eye Hospital	Institutional Review Board of Hayashi Eye Hospital	4-23-35 Hakataekimae, Hakata-ku, Fukuoka-shi, Fukuoka 812-0011, Japan
7	Hyogo Medical University Hospital	Institutional Review Board of Hyogo Medical University Hospital	1-1 Mukogawacho, Nishinomiya-shi, Hyogo 663-8501, Japan
8	Tokyo Medical University Hachioji Medical Center	Tokyo Medical University Hachioji Medical Center Institutional Review Board	1163 Tatemachi, Hachioji-shi, Tokyo 193-0998, Japan
9	Shinshu University Hospital	Shinshu University Hospital Institutional Review Board	3-1-1 Asahi, Matsumoto-shi, Nagano 390-8621, Japan
10	Fukushima Medical University Hospital	Joint Institutional Review Board of Tohoku Clinical Research Organization	1 Hikarigaoka, Fukushima-shi, Fukushima 960-1295, Japan
11	Matsuyama Red Cross Hospital	Institutional Review Board of Matsuyama Red Cross Hospital	1 Bunkyo-cho, Matsuyama-shi, Ehime 790-8524, Japan
12	Miyake Eye Hospital	Yokohama Minoru Clinic Institutional Review Board	3-14-20 Ozone, Kita-ku, Nagoya-shi, Aichi 462-0825, Japan
13	Jichi Medical University Hospital	Jichi Medical University Hospital Institutional Review Board	3311-1 Yakushiji, Shimotsuke-shi, Tochigi 329-0498, Japan

14	University of Miyazaki Hospital	University of Miyazaki Hospital Institutional Review Board	5200 Kiyotakecho, Kihara, Miyazaki- shi, Miyazaki 889-1692, Japan
15	Souseikai Sumida Hospital	Hakata Clinic Institutional Review Board	1-29-1 Honjo, Sumida-ku, Tokyo 130-0004, Japan
16	Rakuwakai Otowa Hospital	Rakuwakai Otowa Hospital Institutional Review Board	2 Otowachinji-cho, Yamashina-ku, Kyoto-shi, Kyoto 607-8062, Japan
