# Supplementary material for: Phase I Study of Tivozanib Eye Drops in Healthy Volunteers and Patients with Neovascular Age-Related Macular Degeneration
Source: Ophthalmol Sci. 2024 May 22;4(6):100553. doi: 10.1016/j.xops.2024.100553 (PMC11331923; doi:10.1016/j.xops.2024.100553)
Supplement: Supplemental Table 2 [file mmc2.pdf]

Table S2. Summary of Adverse Events and Ocular and Other Adverse Events in Cohort 1

Adverse Events	Placebo n = 10		Tivozanib Step 1 0.15 mg/day Japanese with* 0.5 w/v% n = 6		Tivozanib Step 2 0.3 mg/day Japanese with* 1.0 w/v% n = 6		Tivozanib Step 3 0.6 mg/day Japanese with* 1.0 w/v% n = 6		Tivozanib Step 4 0.6 mg/day White with* 1.0 w/v% n = 6		Tivozanib Step 5 0.6 mg/day Japanese with* 2.0 w/v% n = 6		Tivozanib Total N = 30	
	n	(%)	n	(%)	n	(%)	n	(%)	n	(%)	n	(%)	n	(%)
Any TEAE	0		1	(16.7)	3	(50.0)	4	(66.7)	0		3	(50.0)	11	(36.7)
Death	0		0		0		0		0		0		0	
Other serious	0		0		0		0		0		0		0	
Other significant	0		0		0		0		0		0		0	
Any drug-related TEAE	0		0		2	(33.3)	2	(33.3)	0		3	(50.0)	7	(23.3)
Death	0		0		0		0		0		0		0	
Other serious	0		0		0		0		0		0		0	
Other significant	0		0		0		0		0		0		0	
Any TEAE of the study eye	0		0		2	(33.3)	2	(33.3)	0		3	(50.0)	7	(23.3)
Any drug-related TEAE of the study eye	0		0		2	(33.3)	2	(33.3)	0		3	(50.0)	7	(23.3)
**Ocular adverse events in the study eye**														
Eye disorders	0		0		2	(33.3)	2	(33.3)	0		3	(50.0)	7	(23.3)
Eye irritation	0		0		2	(33.3)	2	(33.3)	0		3	(50.0)	7	(23.3)
Foreign body sensation in eyes	0		0		0		1	(16.7)	0		0		1	(3.3)
**Other adverse events**														
General disorders and administration site conditions	0		0		0		1	(16.7)	0		0		1	(3.3)
Pyrexia	0		0		0		1	(16.7)	0		0		1	(3.3)
Investigations	0		1	(16.7)	1	(16.7)	1	(16.7)	0		0		3	(10.0)
Blood triglycerides increased	0		1	(16.7)	0		1	(16.7)	0		0		2	(6.7)
Blood pressure increased	0		0		1	(16.7)	0		0		0		1	(3.3)
Hepatic enzyme increased	0		0		0		1	(16.7)	0		0		1	(3.3)
Nervous system disorders	0		0		0		1	(16.7)	0		0		1	(3.3)
Headache	0		0		0		1	(16.7)	0		0		1	(3.3)

*With nasolacrimal duct occlusion or eyelid closure.

TEAE = treatment-emergent adverse event
