# Supplementary material for: Phase I Study of Tivozanib Eye Drops in Healthy Volunteers and Patients with Neovascular Age-Related Macular Degeneration
Source: Ophthalmol Sci. 2024 May 22;4(6):100553. doi: 10.1016/j.xops.2024.100553 (PMC11331923; doi:10.1016/j.xops.2024.100553)
Supplement: Supplemental Table 3 [file mmc3.pdf]

Table S3. Adverse Events Other Than Ocular Adverse Events in Cohort 2

			**Tivozanib**														
**Adverse Events**	**Placebo n = 12**		**Step 1 0.45 mg/day Japanese with* 0.5 w/v% (TID) n = 6**		**Step 2 0.9 mg/day Japanese with* 1.0 w/v% (TID) n = 6**		**Step 3 1.8 mg/day Japanese with* 1.0 w/v% (TID) n = 6**		**Step 4 1.8 mg/day Japanese without† 1.0 w/v% (TID) n = 6**		**Step 5 1.8 mg/day White with* 1.0 w/v% (TID) n = 6**		**Step 6 1.8 mg/day Japanese with* 2.0 w/v% (TID) n = 6**		**Total n = 36**	
	n	(%)	n	(%)	n	(%)	n	(%)	n	(%)	n	(%)	n	(%)	n	(%)
**Adverse events other than ocular adverse events)**																
Cardiac disorders	0		0		0		1	(16.7)	0		0		0		1	(2.8)
Supraventricular extrasystoles	0		0		0		1	(16.7)	0		0		0		1	(2.8)
Gastrointestinal disorders	0		1	(16.7)	0		0		0		0		0		1	(2.8)
Aphthous ulcer	0		1	(16.7)	0		0		0		0		0		1	(2.8)
Infections and infestations	0		0		0		0		0		1	(16.7)	0		1	(2.8)
Upper respiratory tract infection	0		0		0		0		0		1	(16.7)	0		1	(2.8)
Investigations	2	(16.7)	0		0		1	(16.7)	2	(33.3)	1	(16.7)	0		4	(11.1)
C-reactive protein increased	0		0		0		1	(16.7)	0		1	(16.7)	0		2	(5.6)
White blood cell count increased	0		0		0		1	(16.7)	0		1	(16.7)	0		2	(5.6)
Alanine aminotransferase increased	1	(8.3)	0		0		0		0		0		0		0	
Blood creatine phosphokinase increased	0		0		0		0		1	(16.7)	0		0		1	(2.8)
Blood triglycerides increased	0		0		0		0		1	(16.7)	0		0		1	(2.8)
Blood uric acid increased	1	(8.3)	0		0		0		0		0		0		0	
Musculoskeletal and connective tissue disorders	1	(8.3)	0		0		0		0		0		1	(16.7)	1	(2.8)
Muscle spasms	1	(8.3)	0		0		0		0		0		0		0	
Myalgia	0		0		0		0		0		0		1	(16.7)	1	(2.8)
Nervous system disorders	0		0		0		0		0		1	(16.7)	0		1	(2.8)
Headache	0		0		0		0		0		1	(16.7)	0		1	(2.8)
Respiratory, thoracic, and mediastinal disorders	0		0		0		1	(16.7)	1	(16.7)	0		0		2	(5.6)
Epistaxis	0		0		0		1	(16.7)	0		0		0		1	(2.8)
Oropharyngeal pain	0		0		0		0		1	(16.7)	0		0		1	(2.8)

*With nasolacrimal duct occlusion or eyelid closure.
[†]Without nasolacrimal duct occlusion or eyelid closure.
TID = 3 times daily.
