# Supplementary material for: Phase I Study of Tivozanib Eye Drops in Healthy Volunteers and Patients with Neovascular Age-Related Macular Degeneration
Source: Ophthalmol Sci. 2024 May 22;4(6):100553. doi: 10.1016/j.xops.2024.100553 (PMC11331923; doi:10.1016/j.xops.2024.100553)
Supplement: Supplemental Table 4 [file mmc4.pdf]

Table S4. Individual Participant Visual Acuity Assessed by the Landolt Ring Test for Healthy Volunteers in Cohort 1

Cohort 1	Baseline VA	VA at Day 2	VA at Day 3	VA at Day 5	VA at Day 9	VA at Day 16	VA at Day 23
Placebo							
1	2.0	2.0	2.0	2.0	1.5	1.5	2.0
2	1.5	1.5	1.5	1.5	1.5	1.2	1.5
3	1.5	1.5	1.5	1.5	2.0	2.0	1.5
4	2.0	2.0	2.0	2.0	2.0	2.0	1.5
5	2.0	2.0	1.5	1.5	2.0	2.0	1.5
6	1.5	1.2	1.5	1.5	1.5	1.2	1.5
7	1.2	1.2	1.0	1.2	1.2	1.2	1.5
8	0.7	0.8	0.5	0.6	0.6	0.7	0.7
9	1.5	1.5	2.0	1.5	2.0	2.0	2.0
10	1.5	1.5	2.0	2.0	2.0	2.0	2.0
Mean (SD)	1.540 (0.4033)	1.520 (0.3967)	1.550 (0.4972)	1.530 (0.4270)	1.630 (0.4692)	1.580 (0.4826)	1.570 (0.3860)
Tivozanib eye drops							
1	1.2	1.2	1.5	1.5	1.2	1.5	1.5
2	1.5	1.5	1.5	2.0	2.0	2.0	1.5
3	1.2	1.5	1.5	1.5	1.5	1.5	1.5
4	1.5	2.0	2.0	2.0	2.0	2.0	2.0
5	1.5	1.5	1.5	1.5	1.5	1.5	1.5
6	1.5	1.5	1.5	1.5	1.5	1.5	1.5
7	1.5	2.0	1.5	2.0	1.5	2.0	1.5
8	2.0	1.5	1.5	1.5	1.5	1.5	1.5
9	1.5	1.5	1.5	1.5	1.2	1.5	1.5
10	1.5	1.5	1.5	1.5	1.5	1.5	1.5
11	1.5	1.5	1.5	1.5	1.5	1.5	2.0
12	1.5	1.5	1.5	2.0	2.0	2.0	2.0
13	1.2	1.2	1.5	1.2	1.5	1.5	1.5
14	1.5	1.5	1.5	1.5	-	2.0	1.5
15	1.5	1.2	1.5	1.5	1.5	1.5	1.5
16	2.0	1.5	1.5	1.5	1.5	1.5	1.5
17	1.5	1.2	1.2	1.5	1.5	2.0	1.5
18	1.5	1.5	1.5	1.5	1.5	1.5	1.2
19	1.0	1.0	1.0	1.0	1.0	1.2	1.2
20	1.5	1.5	1.5	1.5	1.5	1.5	1.5
21	0.9	1.0	1.2	1.0	0.9	1.2	1.2
22	1.0	1.2	1.2	0.8	1.0	1.0	0.9
23	2.0	2.0	2.0	2.0	2.0	2.0	1.5
24	1.0	0.5	0.6	0.8	0.7	1.0	0.8
25	2.0	1.5	2.0	1.5	2.0	2.0	1.5
26	1.5	1.5	1.5	1.5	1.5	1.5	1.5
27	1.2	1.2	1.5	1.5	1.5	1.5	1.5
28	2.0	1.5	1.5	1.5	1.5	1.5	2.0
29	1.5	1.5	1.5	1.5	1.5	1.5	-
30	1.5	1.5	2.0	2.0	1.5	1.5	2.0
Mean (SD)	1.473 (0.3051)	1.423 (0.3002)	1.490 (0.2833)	1.510 (0.3252)	1.483 (0.3230)	1.580 (0.2929)	1.510 (0.2907)

SD = standard deviation; VA = visual acuity.
