# Supplementary material for: Phase I Study of Tivozanib Eye Drops in Healthy Volunteers and Patients with Neovascular Age-Related Macular Degeneration
Source: Ophthalmol Sci. 2024 May 22;4(6):100553. doi: 10.1016/j.xops.2024.100553 (PMC11331923; doi:10.1016/j.xops.2024.100553)
Supplement: Supplemental Table 5 [file mmc5.pdf]

Table S5. Individual Participant Visual Acuity Assessed by the Landolt Ring Test for Healthy Volunteers in Cohort 2

Cohort 2	Baseline VA	VA at Day 2	VA at Day 4	VA at Day 8	VA at Day 12	VA at Day 16	VA at Day 21	VA at Day 23	VA at Day 29	VA at Day 36	VA at Day 43
Placebo											
1	1.2	1.2	1.5	1.5	1.5	1.2	1.5	1.2	1.2	1.5	1.5
2	1.5	1.5	1.5	1.5	1.5	1.5	1.5	1.5	1.5	1.5	1.5
3	2.0	2.0	2.0	2.0	2.0	2.0	2.0	2.0	2.0	2.0	2.0
4	2.0	2.0	2.0	1.5	1.5	2.0	2.0	2.0	1.5	2.0	2.0
5	1.5	1.5	1.5	1.5	2.0	1.5	2.0	1.5	2.0	1.5	1.5
6	2.0	1.5	2.0	1.5	1.5	1.5	2.0	2.0	2.0	1.5	1.5
7	1.5	1.2	1.2	1.5	1.5	1.5	1.2	1.5	1.5	1.5	1.2
8	1.5	2.0	2.0	2.0	1.5	2.0	1.5	1.5	1.5	1.5	1.5
9	2.0	2.0	2.0	2.0	1.5	2.0	2.0	2.0	2.0	2.0	1.5
10	1.2	1.2	1.0	1.2	1.2	1.0	0.8	1.0	0.8	1.0	0.9
11	1.5	1.5	1.5	2.0	1.5	1.5	1.5	1.5	1.5	1.5	1.5
12	1.5	1.2	1.5	1.5	1.2	1.2	1.2	1.2	1.5	1.2	1.5
Mean (SD)	1.617 (0.3040)	1.567 (0.3447)	1.642 (0.3502)	1.642 (0.2778)	1.533 (0.2462)	1.575 (0.3519)	1.600 (0.4045)	1.575 (0.3519)	1.583 (0.3689)	1.558 (0.3088)	1.508 (0.2937)
Tivozanib eye drops											
1	1.2	1.5	1.5	1.5	1.5	1.5	1.5	1.5	1.5	1.5	1.5
2	1.5	1.5	1.5	1.5	1.5	1.5	1.5	1.5	1.5	1.5	1.5
3	2.0	1.5	1.5	1.5	1.5	1.5	1.5	1.5	1.5	1.5	1.5
4	1.5	1.5	1.5	1.5	1.5	1.5	1.5	1.5	1.5	1.5	1.5
5	1.2	1.5	1.2	2.0	1.5	1.5	1.5	1.5	1.5	2.0	1.5
6	1.5	1.5	1.5	1.5	2.0	1.2	2.0	1.5	1.5	2.0	1.5
7	2.0	1.5	1.5	2.0	1.5	1.5	1.5	1.5	2.0	1.5	1.5
8	1.2	1.5	1.5	1.5	1.5	1.5	1.5	1.5	1.5	1.5	1.5
9	1.5	1.5	1.2	1.5	1.5	1.5	1.5	1.5	1.5	1.5	1.5
10	1.5	1.5	1.5	1.5	1.5	1.5	1.5	2.0	1.5	1.5	1.5
11	1.2	1.2	1.2	1.5	1.5	1.5	1.5	1.5	1.2	1.2	1.2
12	1.0	1.0	1.5	1.5	1.5	1.5	1.5	1.5	1.5	1.5	1.5
13	1.5	1.5	1.5	1.5	1.5	1.5	1.5	2.0	1.5	1.5	1.5
14	2.0	1.5	1.5	1.5	2.0	1.5	1.5	2.0	2.0	2.0	1.5
15	1.5	1.5	1.5	1.2	1.5	1.5	1.2	1.5	1.5	1.5	1.5
16	1.2	1.5	2.0	1.5	1.5	1.5	2.0	2.0	1.5	1.5	1.5
17	1.5	1.2	2.0	1.5	1.5	1.5	1.5	1.5	1.5	1.5	1.5
18	1.5	1.2	1.5	1.5	2.0	1.5	1.2	2.0	2.0	2.0	1.5
19	1.5	1.5	1.5	1.5	1.5	1.5	1.5	1.5	1.5	2.0	1.5
20	1.5	2.0	2.0	2.0	2.0	2.0	2.0	1.5	1.5	1.5	2.0
21	1.5	2.0	2.0	2.0	2.0	2.0	2.0	2.0	1.5	2.0	2.0

22	1.5	2.0	1.5	1.5	1.5	1.5	1.5	2.0	1.5	2.0	1.2
23	2.0	2.0	1.5	1.5	2.0	2.0	2.0	2.0	2.0	2.0	1.5
24	2.0	1.5	1.5	1.5	1.5	1.5	1.2	1.5	1.5	2.0	1.2
25	1.5	1.5	1.5	1.5	1.0	1.5	1.2	1.2	1.5	1.5	1.5
26	1.2	1.5	1.5	1.5	1.2	1.5	1.5	1.5	1.5	1.5	1.5
27	2.0	2.0	2.0	2.0	2.0	2.0	2.0	2.0	2.0	2.0	2.0
28	1.2	1.2	1.5	1.2	1.2	1.5	1.2	1.5	1.2	1.2	1.2
29	1.0	1.0	1.0	1.2	1.2	1.0	1.0	1.5	1.0	1.2	0.9
30	2.0	2.0	2.0	2.0	2.0	2.0	2.0	1.5	2.0	2.0	2.0
31	1.5	1.5	1.5	2.0	1.5	1.5	1.5	1.5	2.0	2.0	2.0
32	1.0	1.5	1.5	1.2	1.2	1.2	1.5	1.5	1.2	1.5	1.2
33	1.5	1.5	1.5	1.5	1.5	1.5	1.5	1.5	1.5	1.5	1.5
34	1.5	1.5	1.5	1.5	1.5	1.5	1.5	1.5	1.5	2.0	1.5
35	1.5	1.5	1.5	1.5	1.5	1.5	1.5	1.5	1.5	1.5	1.5
36	1.5	1.5	1.5	2.0	2.0	2.0	2.0	1.5	1.5	2.0	1.5
Mean (SD)	1.497 (0.2990)	1.522 (0.2587)	1.544 (0.2360)	1.578 (0.2474)	1.578 (0.2748)	1.553 (0.2286)	1.556 (0.2720)	1.617 (0.2299)	1.558 (0.2477)	1.669 (0.2796)	1.511 (0.2423)

SD = standard deviation; VA = visual acuity.
