# Supplementary material for: Phase I Study of Tivozanib Eye Drops in Healthy Volunteers and Patients with Neovascular Age-Related Macular Degeneration
Source: Ophthalmol Sci. 2024 May 22;4(6):100553. doi: 10.1016/j.xops.2024.100553 (PMC11331923; doi:10.1016/j.xops.2024.100553)
Supplement: Supplemental Table 6 [file mmc6.pdf]

Table S6. Adverse Events Other Than Ocular Adverse Events in Cohort 3

Adverse Events	Tivozanib Step 1 0.45 mg/day Japanese with* 0.5 w/v% (TID) n = 7		Step 2 0.9 mg/day Japanese with* 1.0 w/v% (TID) n = 10		Step 3 1.8 mg/day Japanese with* 1.0 w/v% (TID) n = 11		Total n = 28	
	n	(%)	n	(%)	n	(%)	n	(%)
**Adverse events other than ocular adverse events**								
General disorders and administration site conditions	0		0		1	(9.1)	1	(3.6)
Pyrexia	0		0		1	(9.1)	1	(3.6)
Infections and infestations	1	(14.3)	1	(10.0)	2	(18.2)	4	(14.3)
Gingivitis	0		1	(10.0)	0		1	(3.6)
Herpes zoster	0		0		1	(9.1)	1	(3.6)
Nasopharyngitis	1	(14.3)	0		0		1	(3.6)
Otitis media	0		0		1	(9.1)	1	(3.6)
Injury, poisoning, and procedural complications	1	(14.3)	0		0		1	(3.6)
Arthropod sting	1	(14.3)	0		0		1	(3.6)
Nervous system disorders	0		0		1	(9.1)	1	(3.6)
Headache	0		0		1	(9.1)	1	(3.6)

*With nasolacrimal duct occlusion or eyelid closure.
TID = 3 times daily.
