# Supplementary material for: Phase I Study of Tivozanib Eye Drops in Healthy Volunteers and Patients with Neovascular Age-Related Macular Degeneration
Source: Ophthalmol Sci. 2024 May 22;4(6):100553. doi: 10.1016/j.xops.2024.100553 (PMC11331923; doi:10.1016/j.xops.2024.100553)
Supplement: Supplemental Table 7 [file mmc7.pdf]

Table S7. Comparison of Serum Tivozanib Concentrations Between Healthy Japanese Men and Japanese patients with nAMD

Step	Treatment group	Time point	Serum Tivozanib Concentration (ng/mL)			
			Healthy men		Patients with nAMD	
			n	Mean ± SD	n	Mean ± SD
1	0.45 mg/day (TID) Japanese with* 0.5 w/v%	Day 1, Pre	6	BLQ	7	BLQ
		Day 8, Pre	6	5.91 ± 1.57	7	4.08 ± 1.43
		Day 15, Pre†	6	6.48 ± 1.46	7	5.79 ± 1.88
		Day 22, 24 h	6	7.33 ± 1.83	7	5.84 ± 2.12
		Day 43, 528 h	6	0.210 ± 0.314	7	0.666 ± 0.377
2	0.9 mg/day (TID) Japanese with* 1.0 w/v%	Day 1, Pre	6	BLQ	10	BLQ
		Day 8, Pre	6	8.86 ± 2.85	9	10.7 ± 5.6
		Day 15, Pre†	6	11.1 ± 3.8	10	17.4 ± 7.8
		Day 22, 24 h	6	12.8 ± 4.3	10	16.8 ± 7.4
		Day 43, 528 h	6	0.862 ± 0.861	10	1.12 ± 1.06
3	1.8 mg/day (TID) Japanese with* 1.0 w/v%	Day 1, Pre	6	BLQ	11	BLQ
		Day 8, Pre	6	14.0 ± 4.6	11	18.5 ± 13.9
		Day 15, Pre†	6	15.2 ± 5.4	11	23.5 ± 15.2
		Day 22, 24 h	6	16.2 ± 4.7	11	29.2 ± 22.9
		Day 43, 528 h	6	0.742 ± 0.505	10	2.90 ± 3.34

*With nasolacrimal duct occlusion or eyelid closure.

†In healthy men, the result on Day 16 was indicated.

BLQ = below the lower limit of quantification (<0.100 ng/mL); nAMD = neovascular age-related macular degeneration; SD = standard deviation; TID= 3 times daily.
