# Supplementary material for: Phase I Study of Tivozanib Eye Drops in Healthy Volunteers and Patients with Neovascular Age-Related Macular Degeneration
Source: Ophthalmol Sci. 2024 May 22;4(6):100553. doi: 10.1016/j.xops.2024.100553 (PMC11331923; doi:10.1016/j.xops.2024.100553)
Supplement: Supplemental Table 8 [file mmc8.pdf]

Table S8. Individual Patient BCVA as Measured by the ETDRS Visual Acuity Chart for Patients with nAMD in Cohort 3

Cohort 3	Prior anti-VEGF intravitreal injection (Y/N)	Time of last anti-VEGF intravitreal injection prior to Baseline (Day1)	Baseline BCVA	BCVA at Day 8	BCVA at Day 22	BCVA at Day 43
Tivozanib eye drops						
1	N	-	66	67	71	68
2	N	-	83	80	82	78
3	Y	84 months 14 days	65	70	65	54
4	N	-	68	68	69	67
5	N	-	50	55	56	50
6	Y	6 months 23 days	32	43	42	37
7	Y	71 months 2 days	67	69	71	74
8	Y	96 months 5 days	40	46	50	52
9	N	-	71	69	80	85
10	N	-	84	86	85	85
11	N	-	86	-	91	85
12	N	-	87	87	85	88
13	N	-	75	78	79	76
14	N	-	81	78	81	77
15	N	-	82	84	85	84
16	N	-	73	75	80	77
17	Y	4 months 2 days	73	71	67	66
18	N	-	75	74	69	74
19	N	-	75	68	70	70
20	N	-	83	79	85	88
21	N	-	80	86	82	81
22	Y	9 months 18 days to 10 months 17 days	75	80	80	77
23	N	-	54	52	51	58
24	N	-	79	81	80	84
25	N	-	92	90	86	90
26	N	-	84	88	86	-
27	N	-	80	80	80	81
28	N	-	61	60	55	58
Mean (SD)			72.2 (14.18)	72.7 (12.65)	73.7 (12.86)	72.7 (13.61)

Intravitreal injection of anti-VEGF drugs was allowed after the end of the eye drop administration phase (after the end of Day 22).

BCVA = best-corrected visual acuity; ETDRS = Early Treatment Diabetic Retinopathy Study; nAMD = neovascular age-related macular degeneration; SD = standard deviation; VEGF = vascular endothelial growth factor.
